# Immature Surfactant Protein B Increases in the Serum of Patients with Calcific Severe Aortic Stenosis

**DOI:** 10.3390/ijms25126418

**Published:** 2024-06-11

**Authors:** Sonia Eligini, Carlo Savini, Stefania Ghilardi, Alice Mallia, Francesco Vieceli Dalla Sega, Francesca Fortini, Elisa Mikus, Marco Munno, Gloria Modafferi, Piergiuseppe Agostoni, Elena Tremoli, Cristina Banfi

**Affiliations:** 1 Unit of Functional Proteomics, Metabolomics, and Network Analysis, Centro Cardiologico Monzino IRCCS, 20138 Milan, Italy; sonia.eligini@cardiologicomonzino.it (S.E.); stefania.ghilardi@cardiologicomonzino.it (S.G.); alice.mallia@cardiologicomonzino.it (A.M.); marco.munno@cardiologicomonzino.it (M.M.); gloria.modafferi@cardiologicomonzino.it (G.M.); 2 Maria Cecilia Hospital, GVM Care and Research, 48033 Cotignola, Italy; csavini@gvmnet.it (C.S.); fvieceli@gvmnet.it (F.V.D.S.); ffortini@gvmnet.it (F.F.); emikus@gvmnet.it (E.M.); etremoli@gvmnet.it (E.T.); 3 Dipartimento di Scienze Mediche e Chirurgiche, Alma Mater Studiorum, Università di Bologna, 40126 Bologna, Italy; 4 Dipartimento di Biologia e Biotecnologie “Lazzaro Spallanzani”, Università di Pavia, 27100 Pavia, Italy; 5 Heart Failure Unit, Centro Cardiologico Monzino IRCCS, 20138 Milan, Italy; piergiuseppe.agostoni@cardiologicomonzino.it; 6 Department of Clinical and Community Sciences, University of Milan, 20122 Milan, Italy

**Keywords:** valvular disease, surfactant protein B, valve replacement

## Abstract

Valvular disease is a complex pathological condition that impacts countless individuals around the globe. Due to limited treatments, it is crucial to understand its mechanisms to identify new targets. Valve disease may result in pulmonary venous hypertension, which is linked to compromised functioning of the alveolar and capillary membranes and hindered gas exchange. Nonetheless, the correlation between surfactant proteins (SPs) and valve disease remains unexplored. A total of 44 patients were enrolled in this study, with 36 undergoing aortic valve replacement and 8 needing a second aortic valve substitution due to bioprosthetic valve degeneration. Ten healthy subjects were also included. The results showed that patients who underwent both the first valve replacement and the second surgery had significantly higher levels of immature SP-B (proSP-B) compared to control subjects. The levels of the extra-lung collectin SP-D were higher in patients who needed a second surgery due to bioprosthetic valve degeneration, while SP-A levels remained unchanged. The research also showed that there was no reciprocal relationship between inflammation and SP-D as the levels of inflammatory mediators did not differ between groups. The present study demonstrates that circulating proSP-B serves as a reliable marker of alveolar–capillary membrane damage in patients with valvular heart disease.

## 1. Introduction

Valvular heart disease is a severe pathological condition that affects millions of individuals globally, with a prevalence of over 10% in individuals older than 65 in the US and Europe [1,2,3]. There are currently no pharmacological therapies available to prevent, treat, or slow the development of valvular disease in the elderly. Therefore, it is crucial to understand the mechanisms underlying the onset and progression of the disease in order to identify new therapeutic targets. Severe valvular disease is currently treated through surgical intervention (AVR), transcatheter aortic valve replacement (TAVR), or transcatheter aortic valve implantation (TAVI) [4]. However, there is currently no effective medical approach available. It is worth noting that approximately 300,000 artificial valves are implanted worldwide every year, and this number is expected to increase [5].

While not directly linked, valvular disease can affect the alveolar–capillary membrane. Indeed, valve disease may lead to pulmonary venous hypertension, which ultimately leads to alveolar–capillary membrane dysfunction and impaired gas exchange [6,7].

Surfactant proteins (SP)-A, SP-B, SP-C, and SP-D are critical components of the pulmonary surfactant, a complex thin film covering the inner surface of the lung with the function of reducing surface tension at the air–alveolar fluid interface, preventing lung collapse and promoting gas exchange [8]. In addition, surfactant relaxes the airway smooth muscle, prevents lung edema, and exhibits immune function [9].

Several in vitro and in vivo studies have shown the association between the circulating levels of SP-D and cardiovascular diseases [10]. Furthermore, the immature form of surfactant protein B (proSP-B) found in the bloodstream has been suggested as the most reliable marker specific to lung function, indicating dysfunction in the alveolar–capillary membrane and the overall clinical condition of heart failure (HF) [11]. We have previously shown that proSP-B in circulation could serve as a theranostic biomarker, capable of functioning as both a diagnostic and therapeutic indicator [11,12,13].

This study aimed to detect, for the first time, the levels of surfactant proteins in the serum of patients undergoing valve replacement for calcific severe aortic stenosis.

## 2. Results

### 2.1. Characteristics of the Study Participants

Patients undergoing valve replacement for severe calcific aortic stenosis at both the first and second surgery were enrolled in this study.

Demographic and baseline characteristics of the patients are shown in Table 1. The mean age of all patients (aortic valve replacement and redo aortic valve replacement) was 74 years, 48% were women, 88% had hypertension, 70% had dyslipidemia, 22% had diabetes, 9% had an oncological history, 16% had coronary artery disease, and 5% had previous coronary artery bypass graft (CABG) surgery. Regarding the treatment, 84% were taking antihypertensive drugs, 57% statins, 20% antidiabetic drugs, and 14% oral anticoagulants. Eighteen patients were non-smokers and eighteen were either active smokers (*n* = 2) or subjects with a previous history of smoking (n = 16). No information is available regarding when subjects stopped smoking. The characteristics of the patients undergoing the first surgery were similar to those of the patients undergoing a second aortic valve replacement (Table 1). Indeed, there were no significant differences in the patients’ age, gender, hematological profile, LDL levels, presence of hypertension, dyslipidemia, or diabetes. However, in the group of patients who were undergoing aortic valve replacement for the first time, there was a higher percentage of patients taking oral anticoagulants.

### 2.2. Serum Levels of Surfactant Proteins

**Figure 1:**
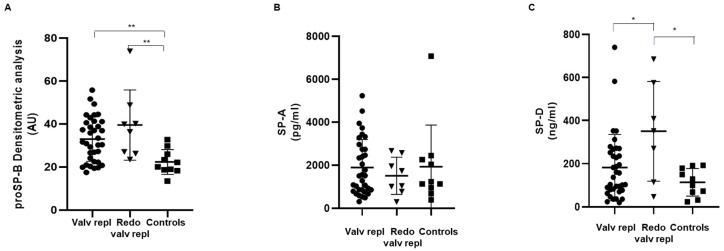
Serum levels of circulating surfactant proteins. (**A**) Surfactant protein proSP-B, (**B**) SP-A, and (**C**) SP-D were detected in serum obtained from patients with valvular disease before valve replacement (Valv repl, n = 35 for proSP-B, n = 34 for SP-A, n = 36 for SP-D), patients requiring a second valve replacement due to a degenerated bioprosthetic valve (Redo valv repl, n = 8 for proSP-B and SP-A, n = 7 for SP-D), and healthy subjects (Controls, n = 10). Data are expressed as mean ± SD. * *p* < 0.05, ** *p* < 0.01 by Mann–Whitney test.

The levels of SPs were measured in the serum obtained from patients and control subjects. As shown in Figure 1A, the levels of immature circulating proSP-B in patients undergoing both the first valve replacement and the second surgery for bioprosthetic valve degeneration were significantly higher than in control subjects, suggesting that the damage in the alveolar–capillary membrane is present in both conditions.

In contrast, the serum levels of SP-A were similar in patients as well as in control subjects, and no significant differences were evidenced (Figure 1B).

SP-D showed a peculiar behavior. In particular, patients who were candidates for the second surgery due to a bioprosthetic valve degeneration showed levels significantly higher than both patients at the first surgery and control subjects (Figure 1C), but no significant difference was observed between patients ready for the first aortic valve surgery and controls.

**Figure 2:**
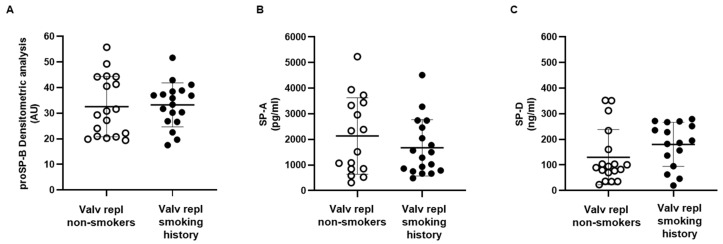
Serum levels of circulating surfactant proteins. (**A**) Surfactant protein proSP-B was detected in serum obtained from non-smoking (n = 18) and smoking and ex-smoking (n = 18) patients with valvular disease before valve replacement. (**B**) Surfactant protein SP-A was measured in serum obtained from non-smoking (n = 16) and smoking and ex-smoking (n = 18) patients with valvular disease before valve replacement. (**C**) Surfactant protein SP-D was detected in serum obtained from non-smoking (n = 18) and ex-smoking (n = 16) patients with valvular disease before valve replacement. Data are expressed as mean ± SD and analyzed by unpaired *t*-test.

We have previously demonstrated that proSP-B is an early and sensitive indicator of the immediate effects of smoke, regardless of potential later oxidative and inflammatory states [14]. In this study, we examined how current or past smoking affects the levels of SPs in patients with aortic valve stenosis. Eighteen patients were non-smokers and 18 were either active smokers (n = 2) or subjects with a previous history of smoking (n = 16). No differences for proSP-B, SP-A, or SP-D were found between non-smokers and subjects with a smoking history, active or previous (Figure 2).

**Figure 3:**
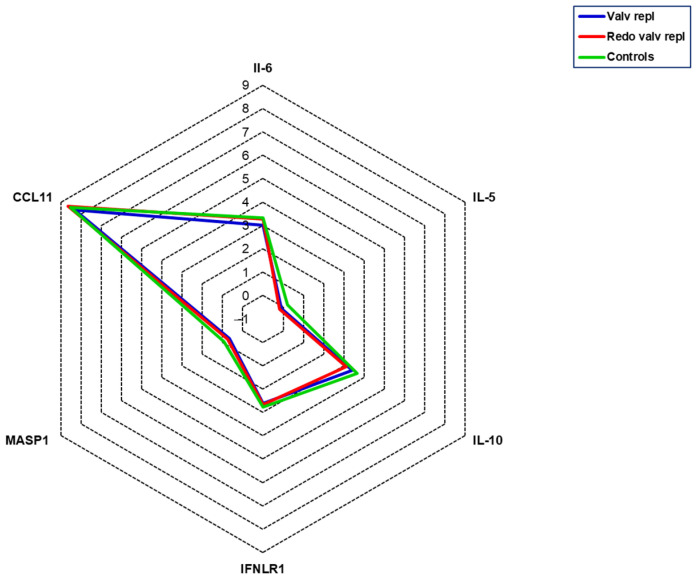
Serum levels of inflammatory mediators. All mediators were measured in serum obtained from patients with valvular disease before valve replacement (Valv repl, n = 36), patients requiring a second valve replacement due to a degenerated bioprosthetic valve (Redo valv repl, n = 7), and healthy subjects (Controls, n = 10). Data are expressed as the mean of NPX.

To assess the presence of an inflammatory state, we measured the levels of interleukin-6 (IL-6), interleukin-5 (IL-5), interleukin-10 (IL-10), interferon lambda receptor 1 (IFNLR1), mannan-binding lectin-associated serine protease 1 (MASP-1), and C-C motif chemokine 11 (CCL11). No significant differences were detected among groups in the current study population for these variables (Figure 3).

## 3. Discussion

This study provides, for the first time, new knowledge about the role of various SPs in severe aortic stenosis. Indeed, we showed that the behavior of proSP-B, SP-A, and SP-D is different in patients with severe aortic stenosis. We have found that only proSP-B is increased in patients with aortic stenosis, while a high value of SP-D is found only in the so-called “redo” cases. These findings are intriguing even if they require further investigation.

The formation of surfactant storage organelles, called lamellar bodies (LBs), postnatal viability, and breathing are dependent only on SP-B among the surfactant proteins [15,16]. The SP-B gene encodes a precursor protein (pre-proSP-B) that includes a signal sequence and three domains with conserved disulfide bridges: N-terminal (SP-BN), middle (SP-BM), and C-terminal (SP-BC) domains [17].

As proSP-B travels to LBs, it goes through a series of protease-mediated processes that convert it into the mature SP-B protein. If you wish to learn more about the biosynthesis and protease-mediated maturation of SP-B, please refer to our previous review [12].

We previously demonstrated that the immature proSP-B, not the mature protein, is related to lung abnormalities, more precisely to the membrane component of DLCO (diffusing capacity of the lung for carbon monoxide) [11,18].

In a larger cohort of stable HF patients, only immature proSP-B and peak VO2 were related to hospitalization, and proSP-B overwhelmed the prognostic role of DLCO or the VE/VCO2 slope, both parameters of ventilatory efficiency being recognized as strong and independent markers of HF prognosis [13].

Considering that in patients with valve disease, SP-B is high prior to operation regardless of whether reintervention is involved or not, it might represent an accurate marker of alveolar damage compared to the other two surfactant proteins. Unfortunately, we do not have DLCO information to reinforce this hypothesis because this exam is considered incongruent with the valve replacement procedure. However, we know that there is a close correlation as stated above.

Further, we demonstrated that proSP-B is a precocious and sensitive index of the acute effects of smoke, independent of the presence of oxidative and inflammatory states that could appear later [14]. However, the increase in SP-B does not seem to be related to smoking because it is not increased in smokers and former smokers. Therefore, it is likely to be related to aortic valve disease, which likely overwhelms the effects of smoking that we previously reported [14].

SP-A and SP-D are members of a superfamily of collagenous, calcium-dependent (C-type) lectins [10,19]. SP-D and SP-A were regarded as lung-specific due to their presence in the phospholipid-rich pulmonary surfactant [20,21,22]. In addition to regulating lipid levels in the pulmonary surfactant, SP-D and SP-A exert antimicrobial effects [23]. In particular, they recognize specific carbohydrate domains in microbes, resulting in complement activation. [19]. These molecules play a crucial role in our innate immune defense by interacting with a variety of microbes. SP-D/-A work in conjunction with alveolar macrophages to facilitate phagocytosis by binding to bacteria, viruses, fungi, and helminthic parasites, marking them for clearance [23]. Furthermore, these proteins have a dual biological activity, meaning they can either suppress or enhance the production of pro-inflammatory cytokines [24,25].

While SP-D expression is mainly concentrated in the lungs, it has also been identified in several non-pulmonary tissues, including the luminal surfaces of the glandular system [26], reproductive tract [27], gastrointestinal tract [26,28], and cardiovascular system [29,30]. Within the cardiovascular system, SP-D expression has been specifically distributed in endothelial and smooth muscle cells. Regarding its role, it has been proposed that it is involved in the modulation of inflammatory signaling. However, we excluded a reciprocal influence between inflammation and SP-D since levels of inflammatory mediators did not vary between groups. The specific origins of circulating SP-D remain incompletely elucidated. It is plausible that SP-D secretion from the arterial wall is a potential source, alongside the primary contribution from lung spillover.

Extra-pulmonary SP-A expression has also been shown. For example, in vitro experiments in murine peritoneal macrophages have highlighted that SP-A deficiency reduced cholesterol accumulation and macrophage foam cell formation. In addition, increased SP-A expression was observed in both human and murine atherosclerotic lesions, and SP-A deficiency reduced atherosclerosis [31]. Recently, research conducted by Kati et al. and Gargiulo et al. has demonstrated a positive correlation between levels of circulatory SP-D and the presence of pulmonary embolism [32], as well as with alveolar leakage in HF [11]. These findings lend support to the hypothesis that fluctuations in circulatory SP-D levels may be partially attributable to lung damage mediated by cardiovascular disease.

Accordingly, recent clinical studies have identified SP-D as a systemic biomarker of cardiovascular morbidity and mortality, indicating its significant role in regulating inflammation and its potential implications for atherosclerosis and cardiovascular diseases [33,34].

In a recent study on elderly twins, researchers found that higher levels of circulating SP-D were associated with an increased risk of mortality [34]. This correlation remained even after adjusting for established risk factors such as smoking, age, sex, plasma cholesterol, and plasma IL-6 levels [33].

Moreover, Hu et al. [35] found a positive association between SP-D levels in the blood, carotid artery wall thickness, and coronary artery calcium buildup.

Further, Brankovic et al. [36] have shown that SP-D is linked to a poorer clinical outcome in chronic HF patients, including worsened heart failure, heart transplantation, and death due to cardiovascular diseases, regardless of clinical profile and medication.

Another study by Otaki et al. [37] demonstrated that patients with high levels of SP-D also had a higher prevalence of diabetes and stenosis in the tibial or peroneal arteries compared to patients with low SP-D levels. This suggests that SP-D in the bloodstream could be a potential target for treatment and a useful marker for tracking the health of arteries in patients with peripheral artery disease (PAD).

In the present study, we observed an increase in SP-D in patients who underwent a second aortic valve replacement procedure. This finding may be related to the persistence of pathological processes other than inflammation and oxidative stress. Indeed, in our previous manuscript, we showed that patients undergoing a second valve replacement showed levels of thiolated albumin (HSA-Cys), a marker of oxidative stress, free sulfhydryl groups, and total antioxidant activity similar to those of controls [38]. As anticipated, SP-D also plays a crucial role in innate immune defense, and it is now being accepted that the immune system plays a crucial role in pathogenesis and disease continuation [39]. This aspect is under investigation.

In reflecting on our current study, it is important to note that there are certain limitations that should be considered. These limitations include the following: (a) a limited sample size, which may impact the generalizability of our findings; (b) being monocentric, meaning that the study was conducted at a single center, potentially impacting the diversity of the study population; (c) the absence of post-surgical controls, which could affect the ability to draw direct comparisons; and (d) the lack of lung function evaluation, which could provide valuable insights into the overall impact of the studied interventions.

While further validation experiments are required to support our results, this study presents important new information that can be used as the foundation for future research on the topic. We have demonstrated for the first time that the immature form of proSP-B is elevated in patients with severe aortic valve disease, potentially indicating damage to the alveolar–capillary membrane due to prolonged hemodynamic burden. Further investigation of the potential correlation between proSP-B and patient survival on this foundation could be an interesting area to explore.

## 4. Materials and Methods

### 4.1. Study Population

The study was carried out at Maria Cecilia Hospital, Cotignola (RA), Italy. Forty-four patients diagnosed with severe calcific aortic stenosis were enrolled between September 2018 and May 2022. All patients were candidates for aortic valve replacement; among them, 36 were to undergo the procedure for the first time and 8 needed a second aortic valve replacement. Inclusion criteria were as follows: age > 65, calcific severe aortic valve stenosis, and hospitalization for aortic valve replacement or redo aortic valve replacement due to a damaged bioprosthetic valve. Criteria exclusion were as follows: infective endocarditis, rheumatic valve disease, end-stage kidney disease, hematological disorders, hepatic dysfunction (bilirubin > 20 mmol/L, prothrombin > 2.0 ratio), severe renal failure (glomerular filtration rate < 30 mL/min/1.73 m^2^), severe cognitive impairment (Short Portable Mental Status Questionnaire (SPMSQ) < 4), life expectancy < 12 months for non-cardiac causes, and bioprosthetic degeneration caused by endocarditis. Ten healthy subjects (5 males and 5 females, age 49.1 ± 6.8) with neither history of cardiovascular disease nor inflammatory disorders and who had not taken any medication the week prior to the blood draw were enrolled as the control group (Controls). Blood samples were collected before surgery. Samples were allowed to clot for 2 h at room temperature, and serum was obtained after centrifugation at 1500× *g* for 15 min. Serum was stored at −80 °C until analysis.

This study was approved by the institutional Ethics Committee and it was conducted in accordance with the principles of the Declaration of Helsinki. All participants provided written informed consent at the time of enrollment.

### 4.2. Immunoassays of proSP-B, SP-A, and SP-D

The immature forms of SP-B, detectable in three predominant forms with molecular mass ranging from 17 to 42 kDa, were analyzed using Western blotting on serum samples, as previously described [11]. Fifty µg of total protein evaluated by the method of Bradford [40] was separated by one-dimensional SDS-PAGE on 15% polyacrylamide gels using a Tris–Tricine buffer system in no reducing conditions and transferred on a nitrocellulose membrane. Membranes were incubated overnight with the primary antibody direct against SP-B (1:1000 mouse anti-human SP-B, F-2; Santa Cruz Biotechnology, D.B.A. Italia S.R.L. Milan, Italy) and subsequently with a secondary 1:1000 diluted goat anti-rabbit antibody conjugated to horseradish peroxidase (Bio-Rad, Milan, Italy) for 1 h. Bands were visualized using the ECL kit (GE Healthcare, Milan, Italy) and quantified by densitometry using image analysis software (QuantityOne version 4.5.2; Bio-Rad, Milan, Italy). SP-A and SP-D levels were measured using commercially available ELISA kits (BioVendor, Heidelberg, Germany). The intra-assay and inter-assay coefficients of variation for SP-A were <5% and <10%, the limit of detection (LOD) was 0.16 ng/mL, and the cut-off level was 1 ng/mL. For SP-D, the intra-assay and inter-assay coefficients of variation were <3% and <4%, LOD was 0.01 ng/mL, and cut-off level was 1.56 ng/mL.

### 4.3. Measurement of Inflammatory Mediators

Serum inflammatory mediators were assessed by a targeted approach based on the proximity extension assay (PEA) principle using the Olink^®^ technology (Target 96 immune response panel, Uppsala, Sweden). Briefly, the target protein binds to the double oligonucleotide-labeled antibody probe, and the resulting DNA sequence is amplified and quantified using quantitative PCR (qPCR). The number of qPCR cycles is related to the expression of the protein in the sample, shown in log base-2 normalized protein expression (NPX) values.

### 4.4. Statistical Analysis

Statistical evaluation was performed using STATA 17.0 SE (StataCorp LLC, College Station, TX, USA). Continuous variables were expressed as median and interquartile range and compared with the Mann–Whitney test. Categorical variables were expressed as absolute number (n) and frequencies (%) and compared with the chi-squared test or Fisher’s exact test as appropriate. The Kolmogorov–Smirnov test was used to assess the normality of the distribution of SPs. A significant departure from normality was found for SP-A (*p* = 0.0022) and for SP-D (*p* = 0.004), but not for SP-B (*p* > 0.100). Comparisons between groups were performed using unpaired Student’s *t*-tests or the Mann–Whitney test as indicated. All tests were two-sided. A *p*-value ≤ 0.05 was considered statistically significant.

## Figures and Tables

**Table 1:** Characteristics of the patients participating in the study.

	Aortic Valve Replacement	Redo Aortic Valve Replacement	*p*
N	36	8	/
Male gender	18 (50.0)	5 (62.5)	0.701
Female gender	18 (50.0)	3 (37.5)	0.701
Age (years)	78.0 (8.5)	70.5 (14)	0.100
Weight (kg)	75.5 (12.5)	70.0 (43)	0.604
BMI (kg/m^2^)	27.5 (3.7)	29.2 (12.2)	0.784
Hemoglobin (g/dL)	12.7 (2.1)	12.8 (3.8)	0.346
Platelets (×10^9^/L)	196 (80)	157.5 (89)	0.041
Neutrophils (×10^9^/L)	/	6.4 (53.8)	/
Lymphocytes (×10^9^/L)	/	2.0 (17.6)	/
Creatinine (mg/dL)	0.91 (0.40)	1.04 (0.36)	0.640
LDL (mg/dL)	71 (38)	92.5 (40)	0.439
Hypertension (n, %)	33 (91.7)	6 (75.0)	0.219
Dyslipidemia (n, %)	27 (75.0)	5 (62.5)	0.663
Diabetes (n, %)	7 (19.4)	2 (25.0)	0.659
Coronary artery disease (n, %)	6 (16.7)	1 (12.5)	1.000
LVEF (%)	61 (11.0)	55 (32.0)	0.692
Peak aortic gradient (mmHg)	72 (32)	75 (27)	0.737
Mean aortic gradient (mmHg)	43 (15)	48 (25)	0.608
Previous coronary artery bypass graft (n, %)	1 (2.8)	1 (12.5)	0.334
Oncological history (n, %)	3 (8.3)	1 (12.5)	1.000
Direct oral anticoagulants (n, %)	6 (16.7)	0 (0.0)	0.053
Statins (n, %)	21 (58.3)	4 (50.0)	0.710
Antidiabetic drugs (n, %)	7 (19.4)	2 (25.0)	0.659
Antihypertensive drugs (n, %)	32 (88.9)	5 (63.0)	0.100
Years since first surgery	/	8.7 (6.1)	/

Continuous variables were presented as median and interquartile range, while categorical variables were presented as absolute number (n) and frequency (%). The *p*-value was calculated using the Mann–Whitney test for continuous variables, and the chi-squared test or Fisher’s exact test for categorical variables, as appropriate. BMI, body mass index; LDL, low-density lipoprotein; LVEF, left ventricular ejection fraction.

## Data Availability

Data collected in the study will be made available using the data repository Zenodo (https://zenodo.org, accessed on 10 April 2024) with restricted access upon request to direzione.scientifica@cardiologicomonzino.it. Any remaining information can be obtained from the corresponding author upon reasonable request.
